# Is Current Evidence Sufficient to Establish the Efficacy of Botulinum Toxin A in Treating Persistent Dry Eye Disease? A Systematic Review and Meta‐Analysis of Interventional Studies With a Critical Review Using GRADE Tool

**DOI:** 10.1002/hsr2.72338

**Published:** 2026-04-16

**Authors:** Mirsaeed Abdollahi, Farbod Semnani, Hadi Vahedi, Navid Sobhi, Mahdi Mohammadkhani, Amin Nabavi, Ali Jafarizadeh

**Affiliations:** ^1^ Nikookari Eye Center Tabriz University of Medical Sciences Tabriz Iran; ^2^ National Center for Health Insurance Research Tehran Iran; ^3^ Translational Ophthalmology Research Center, Farabi Eye Hospital Tehran University of Medical Sciences Tehran Iran; ^4^ Department of Ophthalmology, Hamilton Eye Institute University of Tennessee Health Science Center Memphis Tennessee USA

## Abstract

**Background and Aims:**

Standard management of dry eye disease (DED) relies on artificial tears, anti‐inflammatory therapy, and punctal occlusion in selected cases. However, some patients continue to report persistent symptoms. The present article reviewed the efficacy of Botulinum Toxin A (BTX‐A) for DED that remained symptomatic after these interventions, excluding patients with blepharospasm or hemifacial spasm.

**Methods:**

We conducted a comprehensive search of seven databases (PubMed, Embase, Web of Science, Scopus, CENTRAL, Science Direct, and ProQuest), supplemented by grey literature searches (ProQuest, Scopus, OATD), hand‐searching key ophthalmology journals, and investigating clinical trial registries (ClinicalTrials. gov, ICTRP, ISRCTN). Inclusion criteria were clinical trials and cohort studies assessing BTX‐A in primary DED. Outcomes included tear break‐up time (TBUT), Schirmer's test, and Ocular Surface Disease Index (OSDI). Meta‐analyses utilized STATA 17, combining standardized mean differences (SMD) for TBUT and Schirmer's test, and mean difference (MD) for OSDI. Evidence certainty was evaluated using GRADE.

**Results:**

Out of 5,064 records, five studies (144 eyes) were included. Meta‐analysis indicated no significant improvements in TBUT (Hedges's g = 0.43, 95% CI: −1.34 to 2.20) or Schirmer's test (Hedges's g = 0.23, 95% CI: −0.78 to 1.24). However, OSDI scores showed a significant reduction (MD –14.23; 95% CI: −15.86 to −12.59, *p* < 0.001), with moderate‐certainty evidence.

**Conclusions:**

BTX‐A injections appear to offer symptom relief in persistent DED, though effects on objective measures remain inconclusive, limited by heterogeneity and low power. While initial safety data is favorable, the small number of methodologically weak studies highlights the need for larger, high‐quality trials to clarify BTX‐A's clinical role.

**PROSPERO Registration:**

CRD420251031877.

## Summary

- Persistent dry eye disease can remain symptomatic despite standard options such as topical anti‐inflammatory agents, tear stimulants, artificial tears, and punctal plugs.
- Botulinum toxin type A has emerged as a potential option because it alters tear dynamics in a way that may improve tear retention and symptom control.
- This review indicates that BTX‐A injections provide moderate‐certainty improvement in patient‐reported symptoms (OSDI) in individuals with persistent DED without coexisting blepharospasm or hemifacial spasm.

**Abbreviations**
BEB: benign essential blepharospasm
BTX‐A: Botulinum Toxin A
CENTRAL: Cochrane Central Register of Controlled Trials
CGRP: calcitonin gene‐related peptide
CIs: confidence intervals
DED: dry eye disease
GRADE: Grading of Recommendations Assessment, Development, and Evaluation
HFS: hemifacial spasm
ICTRP: International Clinical Trials Registry Platform
MD: mean difference
MMP‐9: matrix metalloproteinase‐9
NOS: Newcastle‐Ottawa Scale
OATD: Open Access Theses and Dissertations
OSDI: Ocular Surface Disease Index
PICOS: participants, Intervention, Comparison, Outcome, and Study Design
PRISMA: Preferred Reporting Items for Systematic Reviews and Meta‐Analyses Statement
PROSPERO: International Prospective Register of Systematic Reviews
RCTs: randomized controlled trials
REM: random effects model
REML: restricted maximum likelihood
ROB: risk of bias
SEs: standard errors
SMDs: tandardized mean differences
TBUT: tear break‐up time
WOS: Web of Science

## 1 Introduction

Dry eye disease (DED) is a prevalent disorder affecting between 5% and 50% of the general population. According to the International Dry Eye Workshop II, it is defined as a multifactorial disease resulting in symptoms such as ocular discomfort, pain, and fluctuating vision [1].

Even with the availability of several therapeutic approaches, including topical anti‐inflammatory medications, tear stimulants, artificial tears, and punctal plugs, DED may remain intractable [2, 3, 4]. Among emerging therapeutic alternatives, botulinum toxin type A (BTX‐A) has drawn attention due to its ability to modify tear dynamics, which may help retain tears on the surface of the eye and relieve the symptoms [5, 6].

Recent meta‐analyses demonstrated the efficacy of botulinum toxin A (BTX‐A) in the treatment of DED [7, 8]. However, they did not exclude patients with benign essential blepharospasm (BEB) or hemifacial spasm (HFS), both of which can substantially affect eyelid mechanics and ocular surface dynamics, thereby contributing to tear film instability [9, 10, 11], and may influence the interpretation of treatment effects in DED populations. Additionally, subsequent methodological discussions have highlighted potential concerns regarding data extraction consistency across outcomes (e.g., TBUT, Schirmer test, TMH, and OSDI), as well as the importance of clearly defining question frameworks (e.g., PICO) to ensure appropriate study selection and outcome synthesis [12, 13]. Furthermore, while previous systematic reviews had limitations in comprehensive literature search strategies (including grey literature, conference proceedings, key journals, clinical trial registries, or utilizing thesaurus systems to capture studies indexed under alternative terminologies or trade names for BTX‐A), it has also been emphasized that the choice between fixed‐ and random‐effects models should be guided by methodological heterogeneity rather than relying solely on statistical heterogeneity (I²), which may affect overall estimates [14]. In addition, to assess the certainty and strength of evidence in clinical practice, we used the Grading of Recommendations Assessment, Development, and Evaluation (GRADE) tool. Finally, we increased the number of independent assessors at each stage of the review process to minimize the risk of false study exclusion and errors.

Therefore, this systematic review aims to critically evaluate and synthesize existing evidence through meta‐analysis to determine the effectiveness of botulinum toxin A (BTX‐A) in the treatment of patients with persistent dry eye disease.

## 2 Materials and Methods

This study was designed and conducted in accordance with the Cochrane Handbook for Systematic Reviews [15, 16] and was reported according to the Preferred Reporting Items for Systematic Reviews and Meta‐Analyses Statement (PRISMA)−2020 [17] (File S1).

### 2.1 Protocol and Registration

The study protocol was registered in the International Prospective Register of Systematic Reviews (PROSPERO) online database. The registration number is CRD420251031877.

### 2.2 Information Source and Search Strategy

To build the search strategy, we used two thesaurus systems, MeSH terms and Emtree, and by consultation with experts, we made free‐text keywords. A comprehensive search was performed on 7 big databases by Mirsaeed Abdollahi, including PubMed, Embase, Web of Science (WOS), Scopus, Cochrane Central Register of Controlled Trials (CENTRAL), Science Direct, and ProQuest. Furthermore, as grey literature, we searched Scopus, ProQuest, and Open Access Theses and Dissertations (OATD) to identify additional documents. Moreover, we performed a hand‐search on two key journals, “Ophthalmic Plastic and Reconstructive Surgery” and “The Ocular Surface”. To identify unpublished studies, we searched the National Institute of Health Clinical Trials Register (https://clinicaltrials.gov/), the International Clinical Trials Registry Platform (ICTRP), and the UK's clinical study registry (ISRCTN). Finally, references of included studies were hand‐searched for additional relevant studies. All searches were performed manually from January 1990 to January 2025 via their official websites. No restrictions based on language, geographical area, race, or age were applied. The full search syntax for different databases Table S1 (Supporting file 2).

### 2.3 Eligibility Criteria

The study selection was based on the participants, Intervention, Comparison, Outcome, and Study Design (PICOS) framework. All intervention arms of any type of clinical trials and interventional cohort studies (Study design) in participants with persistent DED (Population), assessing the BTX‐A injection efficacy (intervention) in the management of DED (Outcome), compared to before intervention (Comparison). Given the absence of a single accepted standard intervention for persistent DED, the variability of control groups (e.g., sham or alternative treatments), and the primary focus of this study on the efficacy of BTX‐A, only the BTX‐A arm from each study was included in the analysis. The exclusion criteria were patients with eyelid abnormalities, blepharospasm, facial palsy, and unrelated ocular surface disease.

### 2.4 Selection Process

The titles and abstracts of studies were screened by Farbod Semnani and Mirsaeed Abdollahi to identify any relevant studies. Afterward, the full‐text assessment of the studies was performed independently by four researchers, Mirsaeed Abdollahi, Hadi Vahedi, Ali Jafarizadeh, and Farbod Semnani, to determine eligibility for inclusion. Disagreements were resolved through consensus.

### 2.5 Risk of Bias Assessment

The risk of bias (ROB) was assessed independently by the four authors: Mirsaeed Abdollahi, Hadi Vahedi, Ali Jafarizadeh, and Farbod Semnani, and any discord among authors was resolved through consensus. We used the Cochrane Risk of Bias 1 tool (RoB‐1) for assessing ROB in clinical trials [15], which focuses on five main domains: selection bias (random sequence generation and allocation concealment), performance bias (blinding of participants or personnel), detection bias (blinding of outcome assessment), attrition bias (incomplete outcome data), and selective outcome reporting bias. We graded each item as low, high, or unclear risk of bias. Additionally, the Newcastle‐Ottawa Scale (NOS) tool was used for assessing the ROB in cohort studies [18], which focuses on selection (Representativeness of the exposed cohort, Selection of the non‐exposed cohort, and Ascertainment of exposure), comparability, and outcome (Assessment of outcome, enough long follow‐up, and Adequacy of follow‐up). Then, studies were categorized into very high (0–2 points), high (3–6 points), and low risk of bias (7–9 points). Finally, we generated a graphical representation of the risk of bias assessment results using the “QUADAS” module in STATA 17 [19].

### 2.6 Data Extraction

The data collection process was separately performed by four authors, Mirsaeed Abdollahi, Hadi Vahedi, Ali Jafarizadeh, and Farbod Semnani, and any discrepancies were resolved by discussion and consensus. The designed form for data extraction of studies includes the author's name, year of publication, location, study design/sample size, demographic data, inclusion/exclusion criteria, and assessed outcomes.

### 2.7 Statistical Analysis

Statistical analyses followed recommendations in standard references for meta‐analysis of interventional studies, including the Cochrane Handbook for Systematic Reviews of Interventions and Borenstein et al. [14, 15].

In our meta‐analysis, the main outcomes were the baseline and the last post‐intervention tear break‐up time (TBUT), Schirmer's test, and ocular surface disease index (OSDI) score (the lower the OSDI score, the fewer the ocular symptoms). If the means and standard deviations (SDs) of included studies were not available, we estimated the values by using standard errors (SEs), confidence intervals (CIs), range, or sample size, by employing techniques provided by Wan et al. [20]. Since Bukhari et al. [21] presented the results of the TBUT and Schirmer's test as binary outcomes and one arm inclusion from each study, we reported it separately by calculating the risk ratio (RR). Mekhasingharak et al. [22] included two intervention groups receiving 3.3 U and 2.5 U of BTX‐A, respectively. To avoid the risk of multiplicity, these groups were combined, and the mean values were calculated for subsequent analysis.

The meta‐analyses were performed via the ‘meta’ package in STATA software version 17 (StataCorp, College Station, Texas, USA). The random effects model (REM), as a conservative approach, was employed with the restricted maximum likelihood (REML) method and the Knapp‐Hartung adjustment. We employed the pooled standardized mean difference (SMD) as the effect size to assess the TBUT and Schirmer's test, and it was presented as Hedges’ g due to the pre‐post design of the intervention and to minimize any overestimation resulting from small sample numbers. The SMD was classified as trivial (≤ 0.19), mild (0.2–0.49), moderate (0.5–0.79), or high (≥ 0.80) [23]. To assess the OSDI score changes and interpret the meta‐regression results regarding the magnitude of effect for each factor, we used the mean difference (MD).

The I² statistic was employed to evaluate statistical heterogeneity among studies and classified as 0%–40% indicating non‐significant heterogeneity, 30%–60% indicating moderate heterogeneity, 50%–90% indicating considerable heterogeneity, and 75%–100% indicating a high level of heterogeneity [24].

Meta‐regression was conducted to explore the potential source of heterogeneity and assess the impact of follow‐up period, BTX‐A dosage, and mean participants' age (*p* < 0.1). The power analysis for pooled SMD was performed using the “metapower” package in R 4.4.2 [25].

All hypothesis tests were two‐sided with an a priori significance level of *α* = 0.05 unless otherwise specified.

### 2.8 Sensitivity Analysis

#### 2.8.1 Jackknife Method

A sensitivity analysis utilizing the leave‐one‐out approach was performed to evaluate the potential significant impact of any individual research on the overall results. The possible presence of the small study effect was evaluated using Egger's regression test (*p* < 0.1).

#### 2.8.2 The Best‐Case Scenario

We combined the first post‐intervention measurements as the best‐case scenario, assuming this time point most closely reflected the peak efficacy of BTX‐A in managing persistent DED.

### 2.9 Certainty of Evidence

We used GRADE (Grading of Recommendations, Assessment, Development, and Evaluation) to classify the certainty of evidence [26] into high, moderate, low, and very low categories. The initial grading was determined based on the study design: high for randomized controlled trials, moderate for non‐randomized controlled trials, and low for cohort studies. Afterward, we downgraded the evidence based on four domains: risk of bias, inconsistency, imprecision, and publication bias. In contrast, we upgraded the evidence based on three domains: a large effect size, a dose‐response relationship, and adjustment for confounders.

## 3 Result

### 3.1 Study Selection

**Figure 1:**
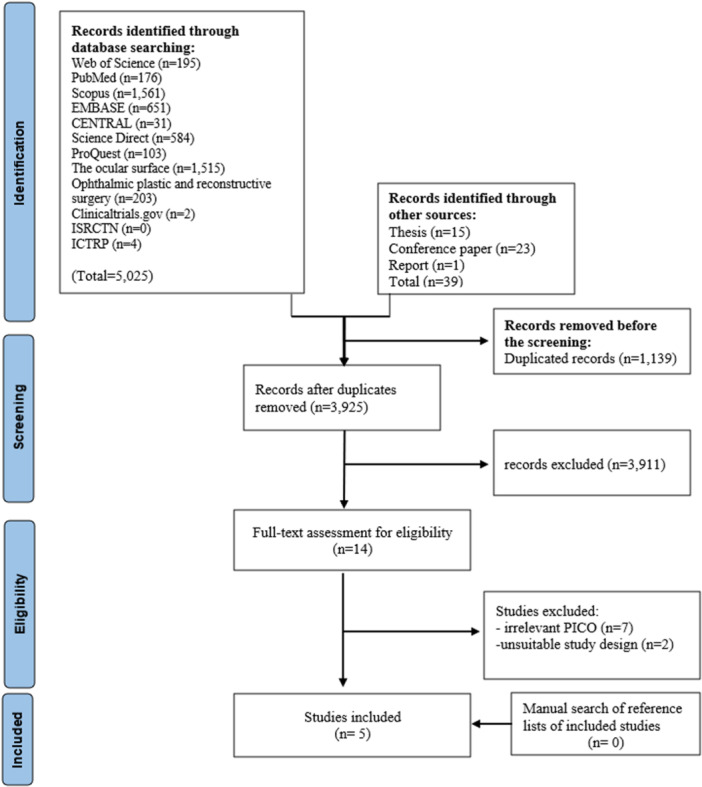
The Preferred Reporting Items for Systematic Reviews and Meta‐Analyses (PRISMA) flow diagram.

The PRISMA flowchart of this study is illustrated in Figure 1. A total of 5,064 records were identified through all sources. Prior to screening, 1,139 studies were removed due to duplications, and 3,925 unique studies were identified. 14 studies were selected for full‐text assessment. Finally, 5 studies met our inclusion criteria and were included in this study [21, 22, 27, 28, 29]. After scanning the references of included studies, no further studies were identified. The list of 9 studies excluded in the full‐text assessment step and the reasons are available in Table S2.

### 3.2 Study Characteristics

Table 1 outlines the key characteristics of the five included studies. Among these, four employed clinical trial designs [21, 22, 28, 29] —including three randomized controlled trials (RCTs), two of which were double‐blinded—while one utilized a retrospective cohort design [27]. All studies were published between 2014 and 2024, collectively involving 172 eyes.

**Table 1:** Study Characteristics.

Study	Country	Design	No. eyes	Mean age	Intervention protocol	Exclusion criteria
Bukhari et al. 2014	Saudi Arabia	Clinical trial	48	47.5	Subcutaneous single dose of 0.1 ml (3.3 IU/0.1 ml), 2 mm inferior to the lid margin, medial to the lower punctum of both lower eyelids	1. Patients who had any history of lacrimal disease, lacrimal surgery, facial palsy, or facial irradiation 2. Those were using any systemic medications that can interfere with tear secretion‐ or drainage‐like chemotherapeutic agents, or ocular medication other than artificial tears at the time of the study
Serna‐Ojeda et al. 2016	Mexico	RCT	20	59.5	Subcutaneous single dose of 4 IU in the medial part of the lower eyelid near the punctum, directed along the medial canthal tendon in one eyelid randomly	1. Eyelid abnormalities (problems with the mechanism of blinking, poor eyelid position, and changes in position or structure of the eyelashes) 2. Those with nasolacrimal obstruction 3. Active corneal infection 4. Severe dry eye cases
Choi et al. 2018	South Korea	Double‐blinded RCT	26	60.2	Subcutaneous single dose of 2.5 IU/0.05 ml, medial fifth region of both upper and lower eyelids, as close as possible to the gray line at the pretarsal orbicularis muscle	1. Eyelid abnormalities (problems with blinking, poor eyelid position, and changes in eyelash position or structure), blepharospasm 2. Active corneal or conjunctival infection 3. History of ocular surgery, including refractive surgery or BTX‐A injection within the past 6 months.
Choi et al. 2021	South Korea	Cohort	56	56.6	Subcutaneous single dose of 2.5 IU injection was given near the punctum and was directed along the medial canthal tendon	—
Mekhasingharak et al. 2024	Thailand	Double‐blinded RCT	22	64.6	Group 1: Subcutaneous single dose of 3.3 IU in the medial part of the lower eyelid, 2 mm inferior to the lid margin, and 5 mm medial to the lower punctum Group 2: Subcutaneous single dose of 2.5 IU in the medial part of the lower eyelid, 2 mm inferior to the lid margin, and 5 mm medial to the lower punctum	1. Patients with dry eye attributable to the eyelid abnormalities 2. Nasolacrimal duct obstruction 3. Active corneal infection 4. History of BTX‐A hypersensitivity

Abbreviations: RCT, Randomized Controlled Trial; IU, International Unit; BTX‐A, Botulinum toxin A

The study populations were demographically diverse, comprising participants of both genders with mean ages ranging from 47.5 to 64.6 years. Before BTX‐A intervention, patients had received conventional treatments such as topical artificial tears, lubricants, or corticosteroids for at least 1 month without significant improvement. The studies applied similar exclusion criteria, which generally included a history of eyelid abnormalities (such as impaired blinking, malposition, blepharospasm, hemifacial spasm, or structural changes in eyelashes), active corneal or conjunctival infections, prior ocular surgeries, or known hypersensitivity to BTX‐A. However, one study excluded patients with severe dry eye cases to avoid possible discomfort or damage [29].

### 3.3 Risk of Bias Assessment

**Figure 2:**
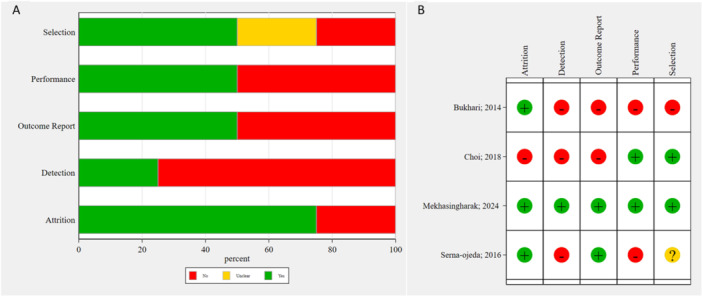
Risk of bias assessment (A presents the overall risk of bias summary across included studies and B presents the detailed risk of bias assessment for each individual study.).

As depicted in Figure 2a, the majority of studies (50%–80%) were assessed as having a high risk of bias in the detection, performance, and outcome reporting domains, reflecting insufficient blinding of participants, personnel, outcome assessors, and reporting of outcomes not pre‐specified in study protocols. Furthermore, ~25% of the studies demonstrated a high risk of bias in the selection, confirming inadequate randomization methods and lack of allocation concealment. In contrast, the attrition bias domain was rated as low risk in the majority of studies (75%), with most providing thorough documentation of participant follow‐up and balanced explanations for any exclusions or missing data. As illustrated in Figure 2b, the study conducted by Mekhasingharak et al. [22] was identified as having the highest methodological quality, exhibiting a low risk of bias across all domains. According to the NOS tool result represented in Table 2, Choi et al. [27] had a high risk of bias, mostly due to the lack of any strategy to adjust for possible confounders.

**Table 2:** Newcastle‐Ottawa score for cohort studies.

Study	Selection				Comparability	Outcome			Total score
	Representativeness of the cases	Selection of the non‐exposed cohort	Ascertainment of exposure	Demonstration that outcome of interest was not present at start of study	The potential confounders were investigated by subgroup analysis or multivariable analysis	Assessment of the outcome	Was follow‐up long enough for outcomes to occur	Adequacy of follow‐up of cohorts	
Choi et al. 2021	*	—	*	*	—	*	*	*	6

### 3.4 Data Synthesis

#### 3.4.1 Tear Break‐Up Time (TBUT)

**Figure 3:**
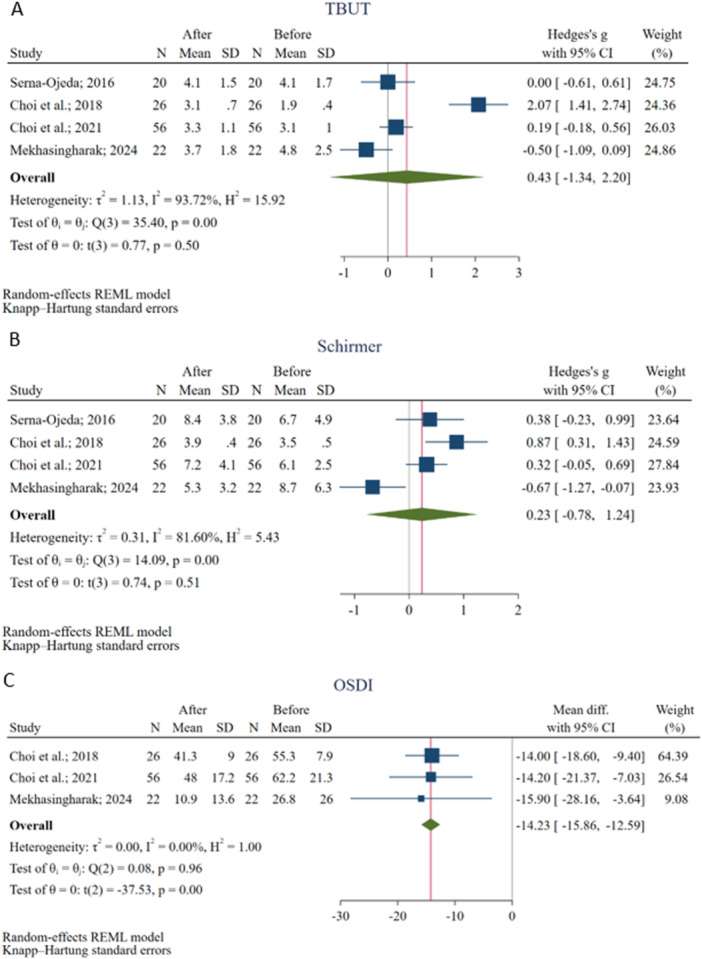
Results of the meta‐analyses for Tear Break‐Up Time (TBUT) (a), Schirmer's Test (b), and the Ocular Surface Disease Index (OSDI) (c).

The meta‐analysis of 4 studies [22, 27, 28, 29] evaluating TBUT changes following BTX‐A injection on 124 eyes revealed no statistically significant overall effect (Hedges's g = 0.43, 95% CI: −1.34 to 2.20). The heterogeneity among the included studies was high (I² = 93.72%), suggesting considerable variability in study outcomes (Figure 3a). Meta‐regression showed that the mean participants' age, measurement time, and BTX‐A dosage could not be the potential source of heterogeneity. Meta‐regression results on variables are available in Table S3.

#### 3.4.2 Schirmer's Test

Similarly, the pooled analysis evaluating Schirmer's test results on 4 studies [22, 27, 28, 29] following BTX‐A injection on 124 eyes indicated no significant improvement in tear production (Hedges's g = 0.23, 95% CI: −0.78 to 1.24) with a high heterogeneity (I² = 81.60%) across studies (Figure 3b). Meta‐regression showed that the mean participants’ age, measurement time, and BTX‐A dosage could not be the potential source of heterogeneity (Table S3).

#### 3.4.3 Ocular Surface Disease Index (OSDI)

Three studies [22, 27, 28] assessed the OSDI score changes following BTX‐A injection on 104 eyes, which showed a significant improvement in symptoms with an overall reduction of 14.23 (95% CI: −15.86 to −12.59, *p* < 0.001) in OSDI score without significant heterogeneity among studies (I² = 0.00%) (Figure 3c). Meta‐regression showed that the mean participants' age, measurement time, and BTX‐A dosage could not be the potential source of heterogeneity (Table S3).

Moreover, Bukhari et al. [21] enrolled 24 eyes to assess the efficacy of BTX‐A and showed that all eyes significantly improved regarding TBUT (RR = 0.52; 95% CI: 0.37–0.72, *p* < 0.001) and Shirmer's test (RR = 0.51; 95% CI: 0.26–0.98, *p* < 0.001).

### 3.5 Sensitivity Analysis

#### 3.5.1 Jackknife Method

**Figure 4:**
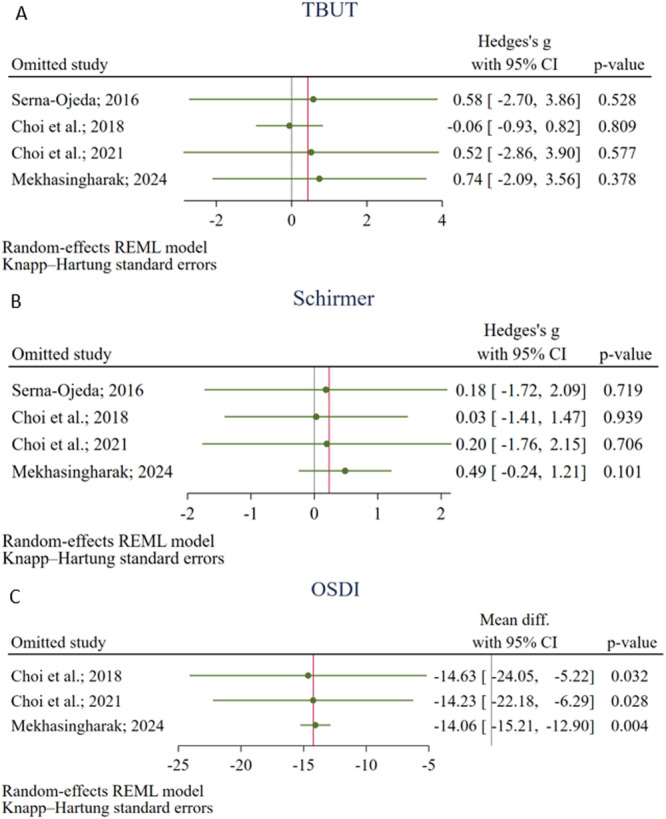
Results of the Jackknife method sensitivity analyses for Tear Break‐Up Time (TBUT) (a), Schirmer's Test (b), and the Ocular Surface Disease Index (OSDI) (c).

The leave‐one‐out method was performed on the results of TBUT, Schirmer's test, and OSDI change scores. There was no outlier study to have a significant influence on the pooled estimates (Figure 4).

#### 3.5.2 The Best‐Case Scenario

**Figure 5:**
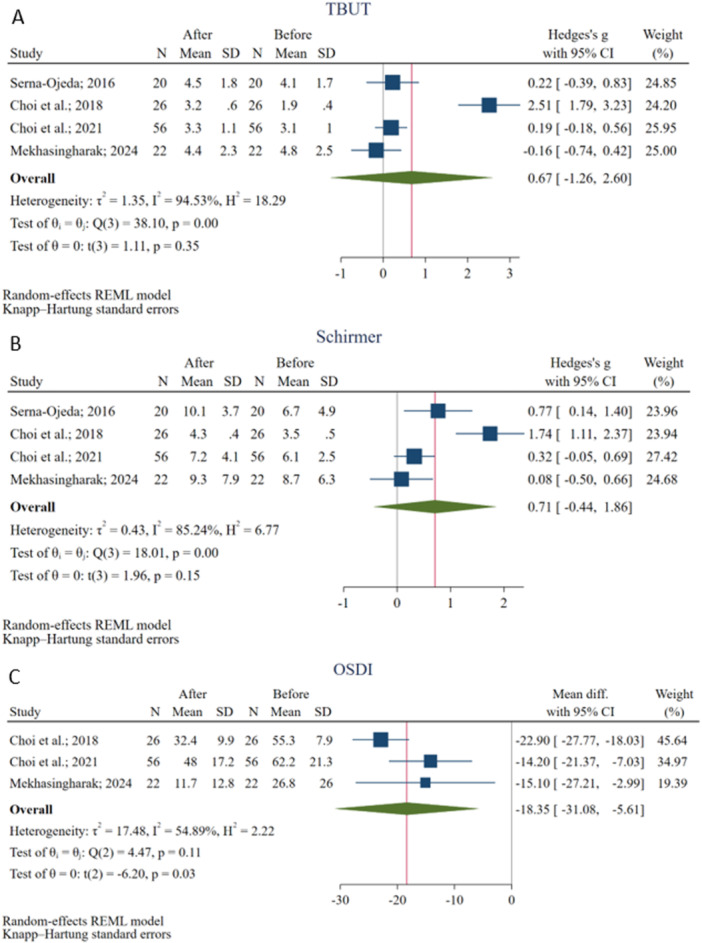
Results of the best‐case scenario sensitivity analyses for Tear Break‐Up Time (TBUT) (a), Schirmer's Test (b), and the Ocular Surface Disease Index (OSDI) (c).

According to this scenario, there was no significant improvement in TBUT (Hedges's g = 0.67; 95% CI: −1.26 to 2.60, I² = 94.53%) and Schirmer's test (Hedges's g = 0.71; 95% CI: −0.44 to 1.86, I² = 85.24%) after BTX‐A intervention. Besides, the OSDI score remained significant (MD = −18.35; 95% CI: −31.08 to −5.61, *p* = 0.03, I² = 54.89%) (Figure 5).

### 3.6 Small Study Effect

The Egger's test showed that there was a low risk of small study effect on the results of TBUT (*p* = 0.61), Schirmer's test (*p* = 0.81), and OSDI change scores (*p* = 0.59).

### 3.7 Power Analysis

Power analysis indicates that the overall statistical power of the meta‐analysis for TBUT and Schirmer's test is 31% and 29%, respectively, indicating a relatively low power status. More studies are needed to achieve adequate power (≥ 80%). However, the meta‐analysis on OSDI demonstrates a high power of 97%.

### 3.8 Certainty of Evidence

The certainty of evidence for the analyzed outcomes, as assessed using the GRADE approach, is summarized in Table 3. The overall certainty of evidence for TBUT and Schirmer's test was rated as low, suggesting limited utility in informing clinical decision‐making. In contrast, the certainty of evidence for the OSDI outcome was considered moderate, indicating its importance in guiding clinical decisions.

**Table 3:** GRADE tool results.

Outcomes	Quality of evidence	Downgrading				Upgrading			Certainty
		ROB	Inconsistency	Imprecision	Publication bias	Effect size	Dose‐response	adjustment	
**TBUT**	⊕⊕⊕⊕ High	Low (0)	Serious (−2)	Moderate (−1)	No (0)	No (0)	Upgraded ( + 1)	No (0)	Limited importance for making a decision
**Schirmer's test**	⊕⊕⊕⊕ High	Low (0)	Serious (−2)	Moderate (−1)	No (0)	No (0)	Upgraded ( + 1)	No (0)	Limited importance for making a decision
**OSDI**	⊕⊕⊕⊕ High	Low (0)	No (0)	Serious (−2)	No (0)	Upgraded ( + 2)	Upgraded ( + 1)	No (0)	Important for making a decision

## 4 Discussion

To the best of our knowledge, this is the first systematic review and meta‐analysis to assess the efficacy of BTX‐A injections in the treatment of persistent DED in patients without eyelid motor disorders, including BEB or HFS. These conditions inherently affect eyelid mechanics and ocular surface dynamics, potentially confounding the assessment of BTX‐A's efficacy in the DED population without underlying neuromuscular disorders [30]. Moreover, the injection site, technique, and dosing of BTX‐A for treatment of the mentioned motor disorders, where higher doses and broader muscle paralysis are required, are vastly different from those for treatment of isolated DED [29]. The results of the current study indicate a notable discrepancy between subjective (patient‐reported) outcomes and objective clinical indices. Accordingly, this analysis revealed a statistically significant and clinically meaningful improvement in OSDI scores following BTX‐A treatment. Nevertheless, no statistically significant overall changes were detected for TBUT or Schirmer's test values. However, the results regarding objective outcomes were inconclusive, as they were either characterized as having low statistical power or high heterogeneity.

A prominent finding of this systematic review is the marked improvement of about 14.23 scores in the OSDI scale, which is higher than the 8.72 [7] and 7.51 [8] improvement in the previous systematic reviews. This could be attributed to the higher baseline OSDI scores in the patients with persistent dry eye, whose symptoms' alleviation could result in a more pronounced reduction in OSDI score (32). The reported I^2^ of zero for OSDI suggests consistency of this larger effect despite the relatively small number of pooled studies. The comparison between the best‐ and worst‐case scenarios indicated that OSDI improvement could be detected approximately from 2 weeks post‐injection, with the effect persisting for at least 4 months after the intervention. Considering this, clinicians may consider initial BTX‐A treatment cycles of 3–6 months for persistent DED with 2.5–4 IU per eyelid, followed by potential symptom‐guided re‐injections. However, this exploratory recommendation may warrant further studies to confirm or refine it.

Nonetheless, there lies the absence of corresponding significant improvements in common objective DED signs like TBUT and Schirmer's test results, contradictory to the previously mentioned systematic reviews. This could be due to the differences in the inclusion criteria (DED vs persistent DED in our study) and the differences in the underlying etiology of DED. BTX‐A injections influence blink mechanics, which can have differing effects in DED with no conspicuous underlying condition (as is the case in the present study) versus secondary DED (a substantial proportion of cases in our study's counterparts), such as that caused by conditions like BEB/HFS. In secondary DED, with abnormal blink patterns, BTX‐A may help normalize blinking and support tear film stability [31, 32]. In contrast, in primary DED, where blink function is typically normal, BTX‐A may disrupt eyelid movement, reduce complete blinking, and increase tear evaporation, potentially counteracting the benefits of reduced tear drainage [33]. On the other hand, the inherent limitations and variability of TBUT and Schirmer's test measurements could contribute to the lack of significant findings for these objective signs [34, 35, 36]. However, the Schirmer's test may not have as good discriminability in milder forms of DED [37, 38]. Even under the best‐case scenario at the time of maximal expected efficacy, no significant improvement was observed in TBUT or Schirmer's test results, reinforcing the negative objective findings.

The subjective‐objective and objective‐objective [22, 27, 39, 40] discordance is not uncommon in DED research and warrants careful consideration. The OSDI captures a broader spectrum of patient experience, including ocular discomfort, functional visual limitations, and environmental triggers, which may not be solely dependent on tear volume or gross tear film stability. The significant symptomatic relief observed could be attributed to BTX‐A functionality beyond sheer modulation of tear volume or stability. BTX‐A could have anti‐inflammatory and neuro‐modulatory characteristics, as Choi et al. [28] showed that BTX‐A injection led to a tear level reduction of the matrix metalloproteinase‐9 (MMP‐9), an inflammatory biomarker, and serotonin, which are involved in pain circuits. BTX‐A can exert similar effects by blocking the release of substance P and calcitonin gene‐related peptide (CGRP), which serve as inflammatory mediators from sensory nerves [41]. Thus, by altering these pathways, BTX‐A could directly alleviate symptoms like pain and irritation, leading to improved OSDI scores without necessarily changing TBUT or Schirmer test values. Captivatingly, the TFOS DEWS II definition encompasses the neurosensory abnormalities in the list of DED etiological factors [1]. The consistent and homogenous improvement in OSDI underscores the importance of patient‐reported outcomes in evaluating DED therapies, particularly for interventions like BTX‐A, where the mechanism of symptomatic relief might be multifactorial.

Despite the exclusive patient population of this review, the meta‐analyses for TBUT and Schirmer's test were marked by extremely high statistical heterogeneity (I^2^ > 90%). The meta‐regression conducted as part of this review, examining mean participant age, measurement time, and BTX‐A dosage, did not identify these factors as significant sources of heterogeneity.

The inability of the meta‐regression to fully explain this substantial variability suggests that other unexplored factors might be involved. These could include subtle differences in baseline DED severity or subtype (e.g., predominantly aqueous‐deficient versus evaporative [3] even within the persistent DED cohort. Variations in BTX‐A intervention details, such as the precise injection technique (depth, number of points, exact anatomical targeting relative to the puncta and thus the level of orbicularis oculi muscle paralysis and variations in blink rate response), specific BTX‐A formulation used, and minor differences in outcome measurement protocols across the included studies, could also contribute significantly [8]. Moreover, concomitant treatments, such as the permitted use of artificial tears (as Serna‐Ojeda et al. [29] allowed them as needed), could modify the observed effects of BTX‐A.

The safety profile of BTX‐A is a of grave importance as well. Considering the five primary studies included in this systematic review, BTX‐A injections for persistent DED were generally well‐tolerated. Adverse events reported were typically mild, transient, and localized, consistent with the known muscle‐paralyzing effects of the toxin. Serna‐Ojeda et al. [29] reported minimal lower lid retraction as the only adverse event, with no problematic lacrimation. Choi MG et al. [28] observed no complications, and Choi EW et al. [27] stated that no subjects complained of specific side effects. Mekhasingharak et al. [22] noted minimal lower lid retraction in a minority of eyes with both 2.5 U and 3.3 U doses and one instance of increased lower eyelid laxity with the higher dose, but no serious adverse events like ectropion or strabismus. Bukhari [21] reported that some patients experienced shampoo getting into their eyes (suggesting mild lagophthalmos) and a lack of improvement in foreign body sensation for a subgroup of patients. The functional disability noted by Bukhari (shampoo in eyes) depicts that even subtle muscle weakening can impact daily activities and should be part of patient counseling. Although, therapeutic peri‐punctal BTX‐A improved symptoms safely in persistent DED, aesthetic may yield short‐term tear film benefits (initial Schirmer increase 0–1 month post‐injection) but long‐term dry eye (more OSDI severity) via reduced blink completeness and TBUT decline at 1–6 months. Clinicians should screen DED patients pre‐aesthetically and counsel on risks, as symptoms often resolve post‐effect (3–6 months) [42].

A key distinguishing feature of the present study is its focused inclusion criteria, specifically excluding patients with BEB or HFS, as to provide a clearer assessment of BTX‐A's efficacy in persistent DED. However, the conclusions of this review are inherently limited by the quantity and quality of the available primary studies. The strict inclusion criteria resulted in the inclusion of only five studies (four clinical trials and one cohort study, totaling 172 eyes for general characteristics). This small number of studies directly contributed to a critical finding of this review: the low statistical power for the TBUT (31%) and Schirmer's test (29%) meta‐analyses. This low power emphasizes a high probability of Type II error, delineating that the absence of a statistically significant effect for these objective outcomes should not be interpreted as definitive evidence of no effect.

Furthermore, the risk of bias assessment (Figure 2a) revealed significant methodological drawbacks. This is also reflected in the GRADE assessment, which rated the certainty of evidence for TBUT and Schirmer's test outcomes as low, while the OSDI outcome was rated as moderate. Broader limitations in the current evidence base for BTX‐A in DED include considerable variability in BTX‐A protocols (dose, injection technique, formulation), small sample sizes in primary studies, and relatively short follow‐up durations, all of which preclude interpreting results in a conclusive manner.

**Figure 6:**
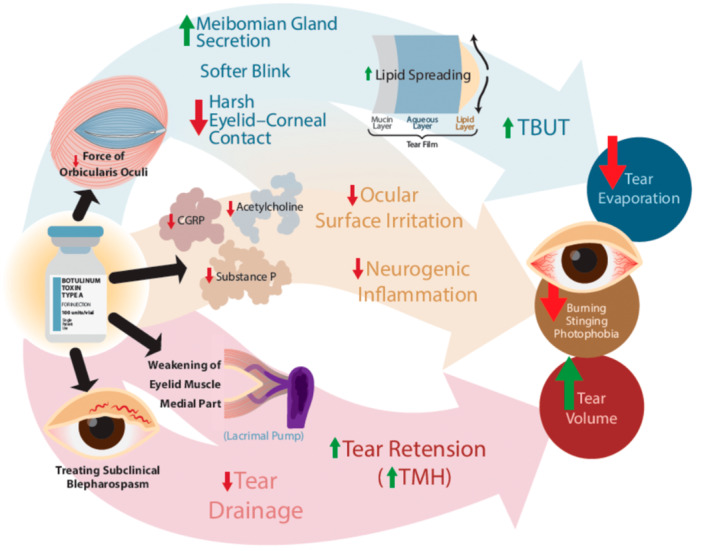
The figure highlights three pathways through which periocular botulinum toxin A can improve dry eye disease, including reduced tear evaporation, attenuation of ocular surface inflammation and irritation, and increased tear volume.

Future research should prioritize large‐scale, multicenter, randomized controlled trials with more robust methodology, to minimize the risks of bias identified in Figure 2a. These trials should preferably focus on DED populations similar to that studied here (i.e., without BEB/HFS) to further clarify BTX‐A's role. Standardization of BTX‐A treatment protocols (dose, injection technique, formulation) is paramount. Longer‐term follow‐up is warranted to assess the durability of effects and optimal re‐injection intervals (Figure 6).

## 5 Conclusion

In conclusion, this systematic review and meta‐analysis provides moderate‐certainty evidence that BTX‐A injections significantly improve patient‐reported symptoms (OSDI) in individuals with persistent DED without concomitant BEB or HFS. However, the effect on objective signs like TBUT and Schirmer's test is inconclusive due to high heterogeneity and low statistical power in this restricted population. While BTX‐A appears generally safe for this indication, the reliability of current evidence is hampered by the small number of studies and methodological shortcomings in some of the primary studies.

## Author Contributions

Conceptualization: Ali Jafarizadeh Methodology: Mirsaeed Abdollahi, Farbod Semnani Software: Navid Sobhi Data curation: Farbod Semnani, Hadi Vahedi Investigation: Mirsaeed Abdollahi, Farbod Semnani, Ali Jafarizadeh Validation: Mirsaeed Abdollahi Formal analysis: Mirsaeed Abdollahi Supervision: Amin Nabavi, Ali Jafarizadeh Visualization: Navid Sobhi Project administration: Ali Jafarizadeh Resources: Ali Jafarizadeh Writing ‐ original draft: Mirsaeed Abdollahi, Farbod Semnani, Hadi Vahedi, Navid Sobhi, Mahdi Mohammadkhani, Ali Jafarizadeh Writing ‐ review & editing: Mirsaeed Abdollahi, Navid Sobhi, Mahdi Mohammadkhani, Amin Nabavi, Ali Jafarizadeh. With their knowledge and criticism, all of the authors contributed to the development of this study. It should be noted that Mirsaeed Abdollahi and Farbod Semnani are co‐first authors of this manuscript. All authors read and approved the final manuscript.

## Funding

The authors have nothing to report.

## Ethics Statement

This study was conducted in accordance with the principles outlined in the Declaration of Helsinki.

## Consent

The authors have nothing to report.

## Conflicts of Interest

The authors declare no conflicts of interest.

## Transparency Statement

The lead author Ali Jafarizadeh affirms that this manuscript is an honest, accurate, and transparent account of the study being reported; that no important aspects of the study have been omitted; and that any discrepancies from the study as planned (and, if relevant, registered) have been explained.

## Supporting information

Supporting File 1

Supporting File 2

## Data Availability

The authors confirm that the data supporting the findings of this study are available within the article and its supplementary materials.
