# Supplementary material for: Is Current Evidence Sufficient to Establish the Efficacy of Botulinum Toxin A in Treating Persistent Dry Eye Disease? A Systematic Review and Meta‐Analysis of Interventional Studies With a Critical Review Using GRADE Tool
Source: Health Sci Rep. 2026 Apr 16;9(4):e72338. doi: 10.1002/hsr2.72338 (PMC13086625; doi:10.1002/hsr2.72338)
Supplement: Supplementary file 2 — Supporting File 2 [file HSR2-9-e72338-s002.docx]

**Supplementary Tables**

Supplementary Table S1 - Search syntax

| Database | Search Syntax | Results |
| --- | --- | --- |
| PubMed | ("botuli* toxin" OR "botuli* neurotoxin” OR "botuli* exotoxin" OR "abobotulinum toxin A" OR "abobotulinumtoxin A" OR "abobotulinumtoxinA" OR "agn 151607" OR "agn151607" OR "alluzience" OR "ant 1207" OR "ant 1401" OR "ant 1403" OR "ant1207" OR "ant1401" OR "ant1403" OR "azzalure" OR "bocouture" OR "boe-tox" OR "BoNT A" OR "BoNT A DS" OR "BoNT serotype A" OR "botox" OR "botox (100 U) injection" OR "botox (oculinum)" OR "botox 100E" OR "botox a" OR "botox cosmetic" OR "botulift" OR "botulin A" OR "botulin toxin a" OR "botulinium a toxin" OR "botulinum a exotoxin" OR "botulinum a toxin" OR "botulinum neurotoxin a" OR "botulinum neurotoxin type A" OR "botulinum toxin type A" OR "botulinum toxins, type A" OR "BTXA" OR "clostridium botulinum a toxin" OR "clostridium botulinum endotoxin" OR "Clostridium botulinum neurotoxin A" OR "Clostridium botulinum neurotoxin type A" OR "clostridium botulinum toxin type a" OR "Clostridium botulinum type A neurotoxin" OR "cnt 52120" OR "cnt52120" OR "cunox" OR "daxibotulinum toxin A" OR "daxibotulinumtoxin A" OR "daxibotulinumtoxin A lanm" OR "daxibotulinumtoxinA" OR "daxibotulinumtoxinA lanm" OR "daxibotulinumtoxinA-lanm" OR "daxxify" OR "dtx 021" OR "dtx021" OR "dwp 450" OR "dwp450" OR "dyslor" OR "dysport" OR "evabotulinum toxin A" OR "evabotulinumtoxin A" OR "evabotulinumtoxinA" OR "evosyal" OR "gemibotulinum toxin A" OR "gemibotulinumtoxin A" OR "gemibotulinumtoxinA" OR "gsk 1358820" OR "gsk1358820" OR "hg 102" OR "hg102" OR "hu 014" OR "hu014" OR "hutox" OR "incobotulinum toxin A" OR "incobotulinumtoxin A" OR "incobotulinumtoxinA" OR "ipn 10200" OR "ipn 59011" OR "ipn10200" OR "ipn59011" OR "jeuveau" OR "letibotulinum toxin A" OR "letibotulinumtoxin A" OR "letibotulinumtoxinA" OR "letibotulinumtoxina wlbg" OR "letibotulinumtoxina-wlbg" OR "letybo" OR "liztox" OR "lp 09" OR "lp09" OR "meditoxin" OR "mt 10107" OR "mt 10109" OR "mt10107" OR "mt10109" OR "nabota" OR "neuronox" OR "nivobotulinum toxin A" OR "nivobotulinumtoxin A" OR "nivobotulinumtoxinA" OR "nt 201" OR "nt201" OR "nuceiva" OR "oculinum" OR "onabotulinum toxin A" OR "onabotulinumtoxin A" OR "onabotulinumtoxinA" OR "onaclostox" OR "pm 12759" OR "pm12759" OR "prabotulinum toxin A" OR "prabotulinumtoxin A" OR "prabotulinumtoxin A xvfs" OR "prabotulinumtoxinA" OR "prabotulinumtoxinA xvfs" OR "prabotulinumtoxinA-xvfs" OR "prosigne" OR "purtox" OR "qm 1114" OR "qm1114" OR "relabotulinum toxin A" OR "relabotulinumtoxin A" OR "relabotulinumtoxinA" OR "reloxin" OR "rt 002" OR "rt002" OR "rtt 150" OR "rtt150" OR "vistabel" OR "vistabex" OR "xeomeen" OR "xeomin" OR "botulinum toxin A")  AND  ("dryness of the eye" OR "eye dryness" OR "dry eye" OR "Keratoconjunctivitis Sicca" OR "Ocular Surface Disease" OR "Ocular Surface dryness" OR "Tear Syndrome" OR "Tear Deficiency")  AND  1990:2024[dp] | **176** |
| Scopus | ALL ("botuli* toxin" OR "botuli* neurotoxin" OR "botuli* exotoxin" OR "abobotulinum toxin A" OR "abobotulinumtoxin A" OR "abobotulinumtoxinA" OR "agn 151607" OR "agn151607" OR "alluzience" OR "ant 1207" OR "ant 1401" OR "ant 1403" OR "ant1207" OR "ant1401" OR "ant1403" OR "azzalure" OR "bocouture" OR "boe-tox" OR "BoNT A" OR "BoNT A DS" OR "BoNT serotype A" OR "botox" OR "botox (100 U) injection" OR "botox (oculinum)" OR "botox 100E" OR "botox a" OR "botox cosmetic" OR "botulift" OR "botulin A" OR "botulin toxin a" OR "botulinium a toxin" OR "botulinum a exotoxin" OR "botulinum a toxin" OR "botulinum neurotoxin a" OR "botulinum neurotoxin type A" OR "botulinum toxin type A" OR "botulinum toxins, type A" OR "BTXA" OR "clostridium botulinum a toxin" OR "clostridium botulinum endotoxin" OR "Clostridium botulinum neurotoxin A" OR "Clostridium botulinum neurotoxin type A" OR "clostridium botulinum toxin type a" OR "Clostridium botulinum type A neurotoxin" OR "cnt 52120" OR "cnt52120" OR "cunox" OR "daxibotulinum toxin A" OR "daxibotulinumtoxin A" OR "daxibotulinumtoxin A lanm" OR "daxibotulinumtoxinA" OR "daxibotulinumtoxinA lanm" OR "daxibotulinumtoxinA-lanm" OR "daxxify" OR "dtx 021" OR "dtx021" OR "dwp 450" OR "dwp450" OR "dyslor" OR "dysport" OR "evabotulinum toxin A" OR "evabotulinumtoxin A" OR "evabotulinumtoxinA" OR "evosyal" OR "gemibotulinum toxin A" OR "gemibotulinumtoxin A" OR "gemibotulinumtoxinA" OR "gsk 1358820" OR "gsk1358820" OR "hg 102" OR "hg102" OR "hu 014" OR "hu014" OR "hutox" OR "incobotulinum toxin A" OR "incobotulinumtoxin A" OR "incobotulinumtoxinA" OR "ipn 10200" OR "ipn 59011" OR "ipn10200" OR "ipn59011" OR "jeuveau" OR "letibotulinum toxin A" OR "letibotulinumtoxin A" OR "letibotulinumtoxinA" OR "letibotulinumtoxina wlbg" OR "letibotulinumtoxina-wlbg" OR "letybo" OR "liztox" OR "lp 09" OR "lp09" OR "meditoxin" OR "mt 10107" OR "mt 10109" OR "mt10107" OR "mt10109" OR "nabota" OR "neuronox" OR "nivobotulinum toxin A" OR "nivobotulinumtoxin A" OR "nivobotulinumtoxinA" OR "nt 201" OR "nt201" OR "nuceiva" OR "oculinum" OR "onabotulinum toxin A" OR "onabotulinumtoxin A" OR "onabotulinumtoxinA" OR "onaclostox" OR "pm 12759" OR "pm12759" OR "prabotulinum toxin A" OR "prabotulinumtoxin A" OR "prabotulinumtoxin A xvfs" OR "prabotulinumtoxinA" OR "prabotulinumtoxinA xvfs" OR "prabotulinumtoxinA-xvfs" OR "prosigne" OR "purtox" OR "qm 1114" OR "qm1114" OR "relabotulinum toxin A" OR "relabotulinumtoxin A" OR "relabotulinumtoxinA" OR "reloxin" OR "rt 002" OR "rt002" OR "rtt 150" OR "rtt150" OR "vistabel" OR "vistabex" OR "xeomeen" OR "xeomin" OR "botulinum toxin A" )  AND  ALL ( "dryness of the eye" OR "eye dryness" OR "dry eye" OR "Keratoconjunctivitis Sicca" OR "Ocular Surface Disease" OR "Ocular Surface dryness" OR "Tear Syndrome" OR "Tear Deficiency")  AND  PUBYEAR > 1989 AND PUBYEAR < 2025 | **1561** |
| Embase | ('botuli* toxin' OR 'botuli* neurotoxin' OR 'botuli* exotoxin' OR 'abobotulinum toxin A' OR 'abobotulinumtoxin A' OR 'abobotulinumtoxinA' OR 'agn 151607' OR 'agn151607' OR 'alluzience' OR 'ant 1207' OR 'ant 1401' OR 'ant 1403' OR 'ant1207' OR 'ant1401' OR 'ant1403' OR 'azzalure' OR 'bocouture' OR 'boe-tox' OR 'BoNT A' OR 'BoNT A DS' OR 'BoNT serotype A' OR 'botox' OR 'botox (100 U) injection' OR 'botox (oculinum)' OR 'botox 100E' OR 'botox a' OR 'botox cosmetic' OR 'botulift' OR 'botulin A' OR 'botulin toxin a' OR 'botulinium a toxin' OR 'botulinum a exotoxin' OR 'botulinum a toxin' OR 'botulinum neurotoxin a' OR 'botulinum neurotoxin type A' OR 'botulinum toxin type A' OR 'botulinum toxins, type A' OR 'BTXA' OR 'clostridium botulinum a toxin' OR 'clostridium botulinum endotoxin' OR 'Clostridium botulinum neurotoxin A' OR 'Clostridium botulinum neurotoxin type A' OR 'clostridium botulinum toxin type a' OR 'Clostridium botulinum type A neurotoxin' OR 'cnt 52120' OR 'cnt52120' OR 'cunox' OR 'daxibotulinum toxin A' OR 'daxibotulinumtoxin A' OR 'daxibotulinumtoxin A lanm' OR 'daxibotulinumtoxinA' OR 'daxibotulinumtoxinA lanm' OR 'daxibotulinumtoxinA-lanm' OR 'daxxify' OR 'dtx 021' OR 'dtx021' OR 'dwp 450' OR 'dwp450' OR 'dyslor' OR 'dysport' OR 'evabotulinum toxin A' OR 'evabotulinumtoxin A' OR 'evabotulinumtoxinA' OR 'evosyal' OR 'gemibotulinum toxin A' OR 'gemibotulinumtoxin A' OR 'gemibotulinumtoxinA' OR 'gsk 1358820' OR 'gsk1358820' OR 'hg 102' OR 'hg102' OR 'hu 014' OR 'hu014' OR 'hutox' OR 'incobotulinum toxin A' OR 'incobotulinumtoxin A' OR 'incobotulinumtoxinA' OR 'ipn 10200' OR 'ipn 59011' OR 'ipn10200' OR 'ipn59011' OR 'jeuveau' OR 'letibotulinum toxin A' OR 'letibotulinumtoxin A' OR 'letibotulinumtoxinA' OR 'letibotulinumtoxina wlbg' OR 'letibotulinumtoxina-wlbg' OR 'letybo' OR 'liztox' OR 'lp 09' OR 'lp09' OR 'meditoxin' OR 'mt 10107' OR 'mt 10109' OR 'mt10107' OR 'mt10109' OR 'nabota' OR 'neuronox' OR 'nivobotulinum toxin A' OR 'nivobotulinumtoxin A' OR 'nivobotulinumtoxinA' OR 'nt 201' OR 'nt201' OR 'nuceiva' OR 'oculinum' OR 'onabotulinum toxin A' OR 'onabotulinumtoxin A' OR 'onabotulinumtoxinA' OR 'onaclostox' OR 'pm 12759' OR 'pm12759' OR 'prabotulinum toxin A' OR 'prabotulinumtoxin A' OR 'prabotulinumtoxin A xvfs' OR 'prabotulinumtoxinA' OR 'prabotulinumtoxinA xvfs' OR 'prabotulinumtoxinA-xvfs' OR 'prosigne' OR 'purtox' OR 'qm 1114' OR 'qm1114' OR 'relabotulinum toxin A' OR 'relabotulinumtoxin A' OR 'relabotulinumtoxinA' OR 'reloxin' OR 'rt 002' OR 'rt002' OR 'rtt 150' OR 'rtt150' OR 'vistabel' OR 'vistabex' OR 'xeomeen' OR 'xeomin' OR 'botulinum toxin A')  AND  ('dryness of the eye' OR 'eye dryness' OR 'dry eye' OR 'Keratoconjunctivitis Sicca' OR 'Ocular Surface Disease' OR 'Ocular Surface dryness' OR 'Tear Syndrome' OR 'Tear Deficiency')  AND  [1990-2024]/py | **651** |
| Web of Science | ALL=("botuli* toxin" OR "botuli* neurotoxin" OR "botuli* exotoxin" OR "abobotulinum toxin A" OR "abobotulinumtoxin A" OR "abobotulinumtoxinA" OR "agn 151607" OR "agn151607" OR "alluzience" OR "ant 1207" OR "ant 1401" OR "ant 1403" OR "ant1207" OR "ant1401" OR "ant1403" OR "azzalure" OR "bocouture" OR "boe-tox" OR "BoNT A" OR "BoNT A DS" OR "BoNT serotype A" OR "botox" OR "botox (100 U) injection" OR "botox (oculinum)" OR "botox 100E" OR "botox a" OR "botox cosmetic" OR "botulift" OR "botulin A" OR "botulin toxin a" OR "botulinium a toxin" OR "botulinum a exotoxin" OR "botulinum a toxin" OR "botulinum neurotoxin a" OR "botulinum neurotoxin type A" OR "botulinum toxin type A" OR "botulinum toxins, type A" OR "BTXA" OR "clostridium botulinum a toxin" OR "clostridium botulinum endotoxin" OR "Clostridium botulinum neurotoxin A" OR "Clostridium botulinum neurotoxin type A" OR "clostridium botulinum toxin type a" OR "Clostridium botulinum type A neurotoxin" OR "cnt 52120" OR "cnt52120" OR "cunox" OR "daxibotulinum toxin A" OR "daxibotulinumtoxin A" OR "daxibotulinumtoxin A lanm" OR "daxibotulinumtoxinA" OR "daxibotulinumtoxinA lanm" OR "daxibotulinumtoxinA-lanm" OR "daxxify" OR "dtx 021" OR "dtx021" OR "dwp 450" OR "dwp450" OR "dyslor" OR "dysport" OR "evabotulinum toxin A" OR "evabotulinumtoxin A" OR "evabotulinumtoxinA" OR "evosyal" OR "gemibotulinum toxin A" OR "gemibotulinumtoxin A" OR "gemibotulinumtoxinA" OR "gsk 1358820" OR "gsk1358820" OR "hg 102" OR "hg102" OR "hu 014" OR "hu014" OR "hutox" OR "incobotulinum toxin A" OR "incobotulinumtoxin A" OR "incobotulinumtoxinA" OR "ipn 10200" OR "ipn 59011" OR "ipn10200" OR "ipn59011" OR "jeuveau" OR "letibotulinum toxin A" OR "letibotulinumtoxin A" OR "letibotulinumtoxinA" OR "letibotulinumtoxina wlbg" OR "letibotulinumtoxina-wlbg" OR "letybo" OR "liztox" OR "lp 09" OR "lp09" OR "meditoxin" OR "mt 10107" OR "mt 10109" OR "mt10107" OR "mt10109" OR "nabota" OR "neuronox" OR "nivobotulinum toxin A" OR "nivobotulinumtoxin A" OR "nivobotulinumtoxinA" OR "nt 201" OR "nt201" OR "nuceiva" OR "oculinum" OR "onabotulinum toxin A" OR "onabotulinumtoxin A" OR "onabotulinumtoxinA" OR "onaclostox" OR "pm 12759" OR "pm12759" OR "prabotulinum toxin A" OR "prabotulinumtoxin A" OR "prabotulinumtoxin A xvfs" OR "prabotulinumtoxinA" OR "prabotulinumtoxinA xvfs" OR "prabotulinumtoxinA-xvfs" OR "prosigne" OR "purtox" OR "qm 1114" OR "qm1114" OR "relabotulinum toxin A" OR "relabotulinumtoxin A" OR "relabotulinumtoxinA" OR "reloxin" OR "rt 002" OR "rt002" OR "rtt 150" OR "rtt150" OR "vistabel" OR "vistabex" OR "xeomeen" OR "xeomin" OR "botulinum toxin A")  AND  ALL=("dryness of the eye" OR "eye dryness" OR "dry eye" OR "Keratoconjunctivitis Sicca" OR "Ocular Surface Disease" OR "Ocular Surface dryness" OR "Tear Syndrome" OR "Tear Deficiency")  AND  PY=(1990-2024) | **195** |
| CENTRAL | ("dryness of the eye" OR "eye dryness" OR "dry eye" OR "Keratoconjunctivitis Sicca" OR "Ocular Surface Disease" OR "Ocular Surface dryness" OR "Tear Syndrome" OR "Tear Deficiency")  AND  (botuli* NEXT toxin OR botuli* NEXT neurotoxin OR botuli* NEXT exotoxin OR "abobotulinum toxin A" OR "abobotulinumtoxin A" OR "abobotulinumtoxinA" OR "agn 151607" OR "agn151607" OR "alluzience" OR "ant 1207" OR "ant 1401" OR "ant 1403" OR "ant1207" OR "ant1401" OR "ant1403" OR "azzalure" OR "bocouture" OR "boe-tox" OR "BoNT A" OR "BoNT A DS" OR "BoNT serotype A" OR "botox" OR "botox (100 U) injection" OR "botox (oculinum)" OR "botox 100E" OR "botox a" OR "botox cosmetic" OR "botulift" OR "botulin A" OR "botulin toxin a" OR "botulinium a toxin" OR "botulinum a exotoxin" OR "botulinum a toxin" OR "botulinum neurotoxin a" OR "botulinum neurotoxin type A" OR "botulinum toxin type A" OR "botulinum toxins, type A" OR "BTXA" OR "clostridium botulinum a toxin" OR "clostridium botulinum endotoxin" OR "Clostridium botulinum neurotoxin A" OR "Clostridium botulinum neurotoxin type A" OR "clostridium botulinum toxin type a" OR "Clostridium botulinum type A neurotoxin" OR "cnt 52120" OR "cnt52120" OR "cunox" OR "daxibotulinum toxin A" OR "daxibotulinumtoxin A" OR "daxibotulinumtoxin A lanm" OR "daxibotulinumtoxinA" OR "daxibotulinumtoxinA lanm" OR "daxibotulinumtoxinA-lanm" OR "daxxify" OR "dtx 021" OR "dtx021" OR "dwp 450" OR "dwp450" OR "dyslor" OR "dysport" OR "evabotulinum toxin A" OR "evabotulinumtoxin A" OR "evabotulinumtoxinA" OR "evosyal" OR "gemibotulinum toxin A" OR "gemibotulinumtoxin A" OR "gemibotulinumtoxinA" OR "gsk 1358820" OR "gsk1358820" OR "hg 102" OR "hg102" OR "hu 014" OR "hu014" OR "hutox" OR "incobotulinum toxin A" OR "incobotulinumtoxin A" OR "incobotulinumtoxinA" OR "ipn 10200" OR "ipn 59011" OR "ipn10200" OR "ipn59011" OR "jeuveau" OR "letibotulinum toxin A" OR "letibotulinumtoxin A" OR "letibotulinumtoxinA" OR "letibotulinumtoxina wlbg" OR "letibotulinumtoxina-wlbg" OR "letybo" OR "liztox" OR "lp 09" OR "lp09" OR "meditoxin" OR "mt 10107" OR "mt 10109" OR "mt10107" OR "mt10109" OR "nabota" OR "neuronox" OR "nivobotulinum toxin A" OR "nivobotulinumtoxin A" OR "nivobotulinumtoxinA" OR "nt 201" OR "nt201" OR "nuceiva" OR "oculinum" OR "onabotulinum toxin A" OR "onabotulinumtoxin A" OR "onabotulinumtoxinA" OR "onaclostox" OR "pm 12759" OR "pm12759" OR "prabotulinum toxin A" OR "prabotulinumtoxin A" OR "prabotulinumtoxin A xvfs" OR "prabotulinumtoxinA" OR "prabotulinumtoxinA xvfs" OR "prabotulinumtoxinA-xvfs" OR "prosigne" OR "purtox" OR "qm 1114" OR "qm1114" OR "relabotulinum toxin A" OR "relabotulinumtoxin A" OR "relabotulinumtoxinA" OR "reloxin" OR "rt 002" OR "rt002" OR "rtt 150" OR "rtt150" OR "vistabel" OR "vistabex" OR "xeomeen" OR "xeomin" OR "botulinum toxin A")  AND  1990:2024 [dp] | **31** |
| Science Direct | ("botuli* toxin" OR "botuli* neurotoxin" OR "botuli* exotoxin" OR "abobotulinum toxin A" OR "abobotulinumtoxin A" OR "abobotulinumtoxinA" OR "agn 151607" OR "agn151607" OR "alluzience" OR "ant 1207" OR "ant 1401" OR "ant 1403" OR "ant1207" OR "ant1401" OR "ant1403" OR "azzalure" OR "bocouture" OR "boe-tox" OR "BoNT A" OR "BoNT A DS" OR "BoNT serotype A" OR "botox" OR "botox (100 U) injection" OR "botox (oculinum)" OR "botox 100E" OR "botox a" OR "botox cosmetic" OR "botulift" OR "botulin A" OR "botulin toxin a" OR "botulinium a toxin" OR "botulinum a exotoxin" OR "botulinum a toxin" OR "botulinum neurotoxin a" OR "botulinum neurotoxin type A" OR "botulinum toxin type A" OR "botulinum toxins, type A" OR "BTXA" OR "clostridium botulinum a toxin" OR "clostridium botulinum endotoxin" OR "Clostridium botulinum neurotoxin A" OR "Clostridium botulinum neurotoxin type A" OR "clostridium botulinum toxin type a" OR "Clostridium botulinum type A neurotoxin" OR "cnt 52120" OR "cnt52120" OR "cunox" OR "daxibotulinum toxin A" OR "daxibotulinumtoxin A" OR "daxibotulinumtoxin A lanm" OR "daxibotulinumtoxinA" OR "daxibotulinumtoxinA lanm" OR "daxibotulinumtoxinA-lanm" OR "daxxify" OR "dtx 021" OR "dtx021" OR "dwp 450" OR "dwp450" OR "dyslor" OR "dysport" OR "evabotulinum toxin A" OR "evabotulinumtoxin A" OR "evabotulinumtoxinA" OR "evosyal" OR "gemibotulinum toxin A" OR "gemibotulinumtoxin A" OR "gemibotulinumtoxinA" OR "gsk 1358820" OR "gsk1358820" OR "hg 102" OR "hg102" OR "hu 014" OR "hu014" OR "hutox" OR "incobotulinum toxin A" OR "incobotulinumtoxin A" OR "incobotulinumtoxinA" OR "ipn 10200" OR "ipn 59011" OR "ipn10200" OR "ipn59011" OR "jeuveau" OR "letibotulinum toxin A" OR "letibotulinumtoxin A" OR "letibotulinumtoxinA" OR "letibotulinumtoxina wlbg" OR "letibotulinumtoxina-wlbg" OR "letybo" OR "liztox" OR "lp 09" OR "lp09" OR "meditoxin" OR "mt 10107" OR "mt 10109" OR "mt10107" OR "mt10109" OR "nabota" OR "neuronox" OR "nivobotulinum toxin A" OR "nivobotulinumtoxin A" OR "nivobotulinumtoxinA" OR "nt 201" OR "nt201" OR "nuceiva" OR "oculinum" OR "onabotulinum toxin A" OR "onabotulinumtoxin A" OR "onabotulinumtoxinA" OR "onaclostox" OR "pm 12759" OR "pm12759" OR "prabotulinum toxin A" OR "prabotulinumtoxin A" OR "prabotulinumtoxin A xvfs" OR "prabotulinumtoxinA" OR "prabotulinumtoxinA xvfs" OR "prabotulinumtoxinA-xvfs" OR "prosigne" OR "purtox" OR "qm 1114" OR "qm1114" OR "relabotulinum toxin A" OR "relabotulinumtoxin A" OR "relabotulinumtoxinA" OR "reloxin" OR "rt 002" OR "rt002" OR "rtt 150" OR "rtt150" OR "vistabel" OR "vistabex" OR "xeomeen" OR "xeomin" OR "botulinum toxin A")  AND  ("dryness of the eye" OR "eye dryness" OR "dry eye" OR "Keratoconjunctivitis Sicca" OR "Ocular Surface Disease" OR "Ocular Surface dryness" OR "Tear Syndrome" OR "Tear Deficiency")  AND  PUBYEAR AFT 1989 AND PUBYEAR BEF 2025 | **584** |
| ProQuest | NOFT("botuli* toxin" OR "botuli* neurotoxin" OR "botuli* exotoxin" OR "abobotulinum toxin A" OR "abobotulinumtoxin A" OR "abobotulinumtoxinA" OR "agn 151607" OR "agn151607" OR "alluzience" OR "ant 1207" OR "ant 1401" OR "ant 1403" OR "ant1207" OR "ant1401" OR "ant1403" OR "azzalure" OR "bocouture" OR "boe-tox" OR "BoNT A" OR "BoNT A DS" OR "BoNT serotype A" OR "botox" OR "botox (100 U) injection" OR "botox (oculinum)" OR "botox 100E" OR "botox a" OR "botox cosmetic" OR "botulift" OR "botulin A" OR "botulin toxin a" OR "botulinium a toxin" OR "botulinum a exotoxin" OR "botulinum a toxin" OR "botulinum neurotoxin a" OR "botulinum neurotoxin type A" OR "botulinum toxin type A" OR "botulinum toxins, type A" OR "BTXA" OR "clostridium botulinum a toxin" OR "clostridium botulinum endotoxin" OR "Clostridium botulinum neurotoxin A" OR "Clostridium botulinum neurotoxin type A" OR "clostridium botulinum toxin type a" OR "Clostridium botulinum type A neurotoxin" OR "cnt 52120" OR "cnt52120" OR "cunox" OR "daxibotulinum toxin A" OR "daxibotulinumtoxin A" OR "daxibotulinumtoxin A lanm" OR "daxibotulinumtoxinA" OR "daxibotulinumtoxinA lanm" OR "daxibotulinumtoxinA-lanm" OR "daxxify" OR "dtx 021" OR "dtx021" OR "dwp 450" OR "dwp450" OR "dyslor" OR "dysport" OR "evabotulinum toxin A" OR "evabotulinumtoxin A" OR "evabotulinumtoxinA" OR "evosyal" OR "gemibotulinum toxin A" OR "gemibotulinumtoxin A" OR "gemibotulinumtoxinA" OR "gsk 1358820" OR "gsk1358820" OR "hg 102" OR "hg102" OR "hu 014" OR "hu014" OR "hutox" OR "incobotulinum toxin A" OR "incobotulinumtoxin A" OR "incobotulinumtoxinA" OR "ipn 10200" OR "ipn 59011" OR "ipn10200" OR "ipn59011" OR "jeuveau" OR "letibotulinum toxin A" OR "letibotulinumtoxin A" OR "letibotulinumtoxinA" OR "letibotulinumtoxina wlbg" OR "letibotulinumtoxina-wlbg" OR "letybo" OR "liztox" OR "lp 09" OR "lp09" OR "meditoxin" OR "mt 10107" OR "mt 10109" OR "mt10107" OR "mt10109" OR "nabota" OR "neuronox" OR "nivobotulinum toxin A" OR "nivobotulinumtoxin A" OR "nivobotulinumtoxinA" OR "nt 201" OR "nt201" OR "nuceiva" OR "oculinum" OR "onabotulinum toxin A" OR "onabotulinumtoxin A" OR "onabotulinumtoxinA" OR "onaclostox" OR "pm 12759" OR "pm12759" OR "prabotulinum toxin A" OR "prabotulinumtoxin A" OR "prabotulinumtoxin A xvfs" OR "prabotulinumtoxinA" OR "prabotulinumtoxinA xvfs" OR "prabotulinumtoxinA-xvfs" OR "prosigne" OR "purtox" OR "qm 1114" OR "qm1114" OR "relabotulinum toxin A" OR "relabotulinumtoxin A" OR "relabotulinumtoxinA" OR "reloxin" OR "rt 002" OR "rt002" OR "rtt 150" OR "rtt150" OR "vistabel" OR "vistabex" OR "xeomeen" OR "xeomin" OR "botulinum toxin A") AND NOFT("dryness of the eye" OR "eye dryness" OR "dry eye" OR "Keratoconjunctivitis Sicca" OR "Ocular Surface Disease" OR "Ocular Surface dryness" OR "Tear Syndrome" OR "Tear Deficiency") | **103** |

Supplementary Table S2 - Excluded studies after full-text assessment

| # | Authors | Title | Reason |
| --- | --- | --- | --- |
| 1 | CHUNG et al. | THE EFFECTS OF BOTULINUM TOXIN TREATMENTS OF FILAMENTARY KERATITIS ASSOCIATED WITH DRY EYE AND CORNEAL OCCLUSION STATES | Irrelevant PICO |
| 2 | Sawaed et al. | The effect of botulinum neurotoxin A injections on meibomian glands and dry eye. | Irrelevant PICO |
| 3 | Costa et al. | Lacrimal film evaluation of patients with facial dystonia during botulinum toxin type A treatment | Irrelevant PICO |
| 4 | Žiak et al. | Effect of Botulinum toxin AÂ application in neuro-ophtalmologic indications on SchirmersÂ test and tears osmolarity. | Irrelevant PICO |
| 5 | Fouda et al. | Comparison Between Botulinum Toxin A Injection and Lacrimal Punctal Plugs for the Control of Post-LASIK Dry Eye Manifestations: A Prospective Study | Irrelevant PICO |
| 6 | Diel et al. | Photophobia and sensations of dryness in patients with migraine occur independent of baseline tear volume and improve following botulinum toxin A injections | Irrelevant PICO |
| 7 | Sahlin et al. | Effect of eyelid botulinum toxin injection on lacrimal drainage | Irrelevant PICO |
| 8 | Diaz et al. | Application of botulinum toxin in Horner's muscle for the treatment of dry eye | Unsuitable study design |
| 9 | Diel et al. | Botulinum Toxin A for the Treatment of Photophobia and Dry Eye | Unsuitable study design |

Supplementary Table S3 - Meta-regression results

| Test | Variable | Beta Coefficient | p-value | Residual I^2^ (%) |
| --- | --- | --- | --- | --- |
| TBUT | Mean age | -0.087 (90% CI; -0.758 to 0.585) | 0.743 | 94.86 |
|  | Measurement time | -0.044 (90% CI; -1.448 to 1.360) | 0.935 | 95.06 |
|  | Dose | -0.675 (90% CI; -3.621 to 2.27) | 0.572 | 94.86 |
| Schirmer | Mean age | -0.126 (90% CI; -0.418 to 0.165) | 0.333 | 76.94 |
|  | Measurement time | 0.011 (90% CI; -0.788 to 0.811) | 0.970 | 86.60 |
|  | Dose | -0.090 (90% CI; -1.955 to 1.774) | 0.900 | 88.18 |
| OSDI | Mean age | -0.0001 (90% CI; -0.792 to 0.792) | 0.999 | 83.48 |
|  | Measurement time | -0.229 (90% CI; -2.497 to 2.038) | 0.638 | 74.68 |
|  | Dose | 0.978 (90% CI; -11.564 to 13.520) | 0.709 | 82.99 |
